# Supplementary material for: A Tiny Viral Protein, SARS-CoV-2-ORF7b: Functional Molecular Mechanisms
Source: Biomolecules. 2024 Apr 30;14(5):541. doi: 10.3390/biom14050541 (PMC11118181; doi:10.3390/biom14050541)
Supplement: Supplementary file 1 [file biomolecules-14-00541-s001.zip › biomolecules-2912454-supplementary updated.pdf]

SUPPLEMENTS

A tiny viral protein, SARS-CoV-2-ORF7b: 2 Functional molecular mechanisms.

Gelsomina Mansueto, Giovanna Fusco and Giovanni Colonna*

* Correspondence: giovanni.colonna@unicampania.it
University of Campania, L. Vanvitelli, 80138 - Naples, Italy

SECTION 1: Data supporting the article.
SECTION 2: Robustness of the study

SECTION I

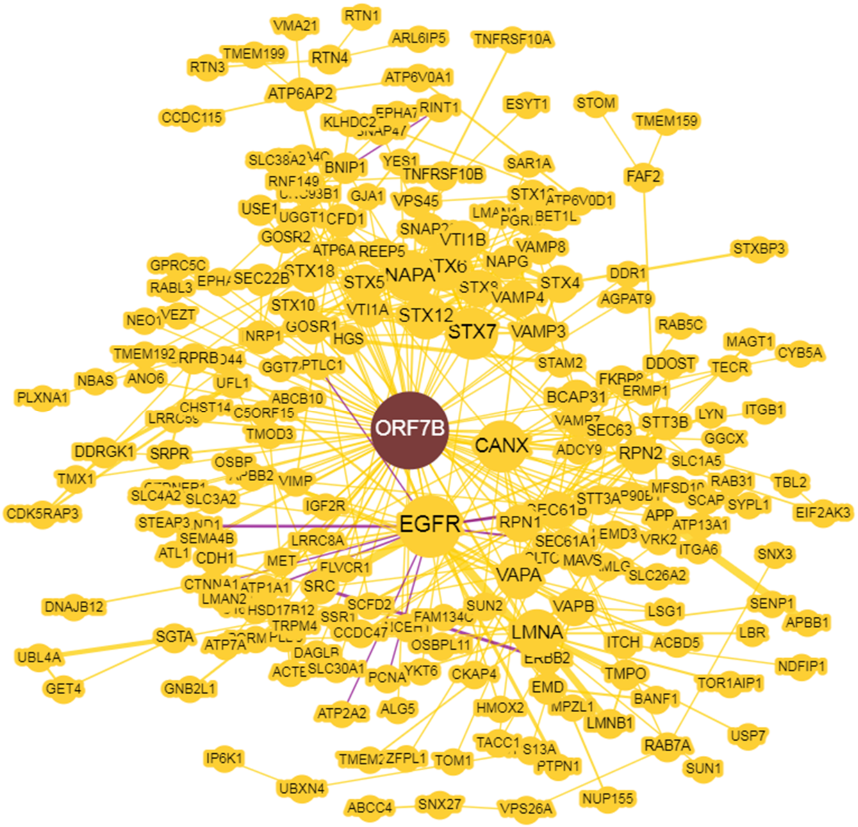

FIGURE S1 – ARBOR representation of ORF7b interactome - ORF7b interactions shown by BioGRID as an ARBOR representation (a dynamic layout that applies physical forces to repel and attract related nodes) using a minimum evidence value of 4 (on a scale from 2 to 28). The minimal evidence selection option of BioGRID

allows you to show/hide edges based on the number of unique curated interactions that reference the association. We have selected the nodes of the innermost layers of figure 1 with the highest degrees using evidence 4. The larger nodes represent the proteins with more edges. Two proteins stand out among all, EGFR and CANX (Calnexin). Human EGFR is a transmembrane receptor member of the protein kinase superfamily. It binds ligands of the EGF family by activating signaling paths to convert extracellular cues into appropriate cellular responses [182]. EGFR is also a component of the cytokine storm, which contributes to a severe form of Coronavirus Disease 2019. In the graph, EGFR appears as an aggregator node associated with all three viral proteins (Fig.1).

Human-CANX encodes a member of the calnexin family of molecular chaperones. It plays a major role in the quality control of protein folding of the ER at the level of endoplasmic reticulum, extracellular exosome, vesicles, mitochondria, and neuronal cell body [183,184].

Table S1. List of 75 proteins of level 6 to 4 present in BioGRID as specific interactors of ORF7b.

ABCB10, ABCC1, ADCY9, AGPAT9, ALG5, ANO6, ATL1, ATP13A1, ATP1A1, ATP6AP1, ATP7A, BCAP31, C16ORF58, C5ORF15, CAMLG, CANX, CTDNEP1, DAGLB, DDR1, EGFR, EPHA2, ERBB2, ERMP1, GGCX, GGT7, GJA1, GOSR1, GPRC5C, HMOX2, HSD17B12, IGF2R, ITGA6, LEMD3, LMAN1, LMAN2, LRRC8A, MFSD10, MPZL1, NCEH1, NEO1, PLD3, RABL3, REEP5, RNF149, SAR1A, SCAP, SEC61A1, SEC61B, SEC63, SEMA4B, SEMA4C, SLC1A5, SLC26A2, SLC30A1, SLC38A2, SLC3A2, SLC4A2, SRPR, STEAP3, STT3A, STX10, STX12, STX16, STX6, STX7, TMEM192, TMX1, TRPM4, UNC93B1, VAMP3, VEZT, VIMP, VPS45, VTI1A, YES1.

Note: After conducting a STRING analysis test, we found minimal functional tendency for the proteins in red to interact, so we removed them. We employed the remaining 51 proteins (highlighted in black) for the present study. Figures 2S and 3S show details and network parameters.

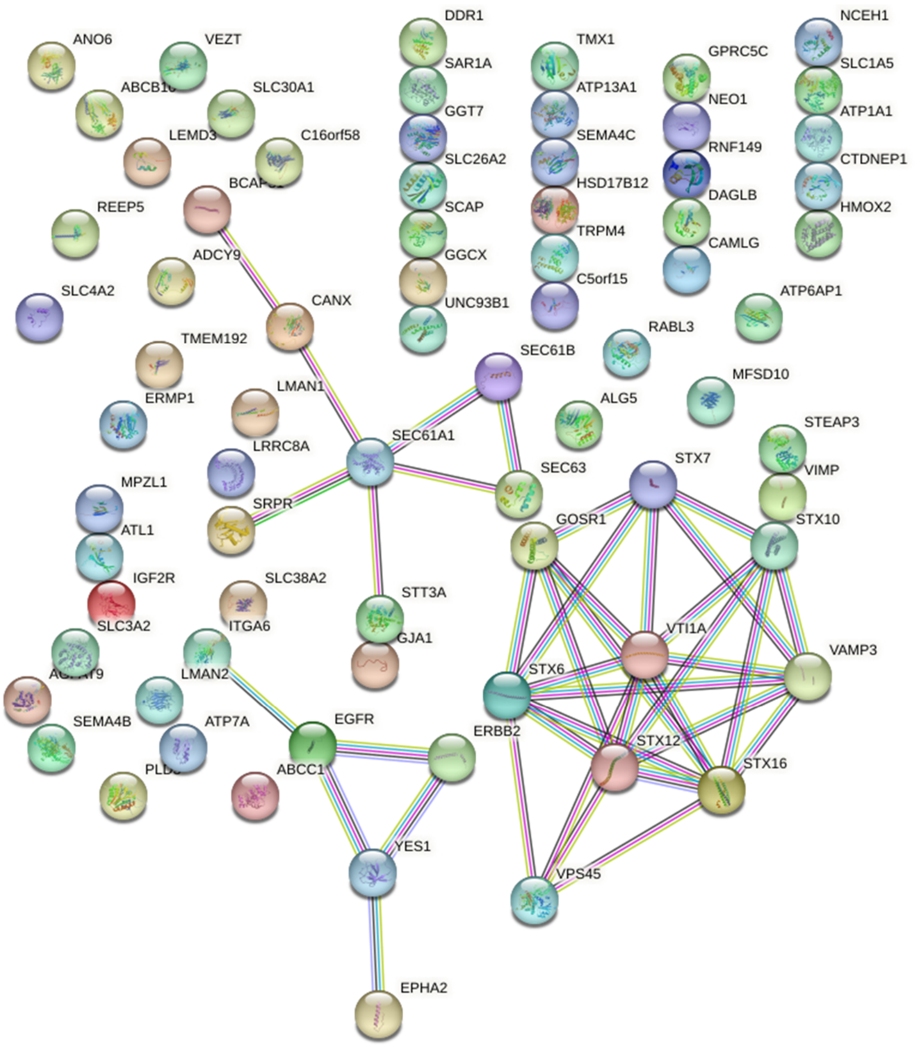

Figure S2 – 75 protein network - Network of the 75 most significant proteins interacting with ORF7b as calculated by STRING. The score used is 0.9 (highest confidence) to select the proteins that have greater statistical significance in the direct functional interactions. Despite using all seven source channels, the net calculation still resulted in many disconnected proteins. All the integrated information used to generate the network comes from the data reported in the international literature or databases. When there is a shortage of publications on a specific topic, it is typical to see a lack of direct and specific information between two nodes in the network generated from the data documented in international literature or databases. Despite appearances the interactome has a p-value <1.0e-16, an average node degree of 1.04, an average local clustering coefficient of 0.209, and 39 edges which are higher than the 3 edges statistically predicted for the same 75 nodes in a random graph. Despite these apparently favorable parameters, the large number of single disconnected nodes, as well as the presence of multiple disconnected modules, would produce unreliable calculated values of the topological parameters.

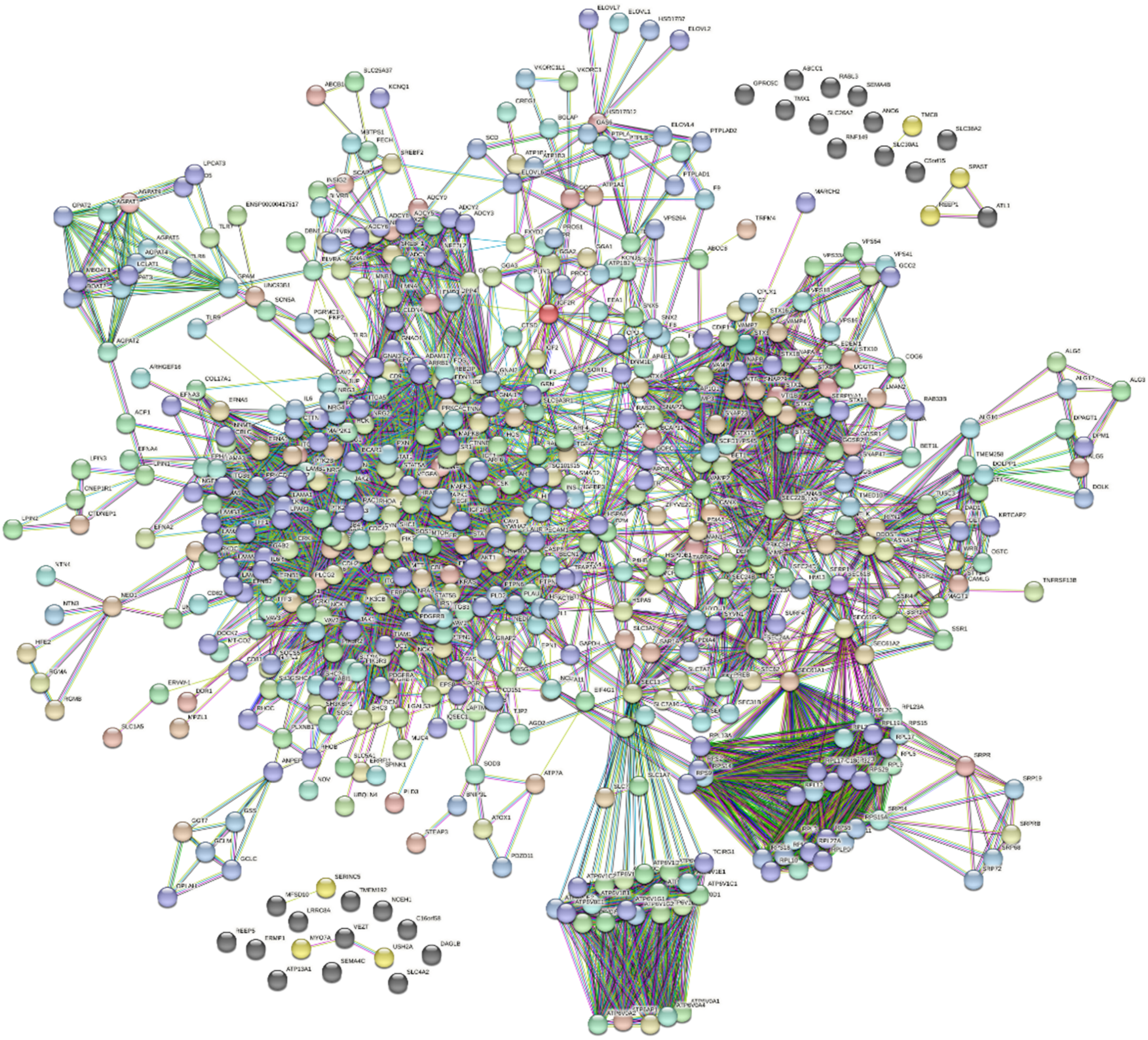

Figure S3 – Functional enrichment of the network (shown in **Figure S2)**.
As 24 parent proteins were still unconnected (clustered in the upper right and lower left corners), we removed them from the initial set. Hence, 51 starting proteins remain. They are the basis for a statistically significant functional enrichment of 500 first order proteins (direct interactors). We show this network and its topological details in **figure 2**. The nodes in yellow are specific interactors of some of the eliminated original proteins. The reasons for pruning are the same as in the previous figure.

Table S2. List of 51 proteins extracted from BioGRID and used on STRING to calculate the human interactome model.

ABCB10, ADCY9, AGPAT9, ALG5, ATP1A1, ATP6AP1, ATP7A, BCAP31, CAMLG, CANX, CTDNEP1, DDR1, EPHA2, ERBB2, GGCX, GGT7, GJA1, GOSR1, HMOX2, HSD17B12, IGF2R, ITGA6, LEMD3, LMAN1, LMAN2, MPZL1, NEO1, PLD3, SAR1A, SCAP, SEC61A1, SEC61B, SEC63, SLC1A5, SLC3A2, SRPR, STEAP3, STT3A, STX10, STX12, STX16, STX6, STX7, TRPM4, UNC93B1, VAMP3, VIMP, VPS45, VTI1A, YES1, EGFR.

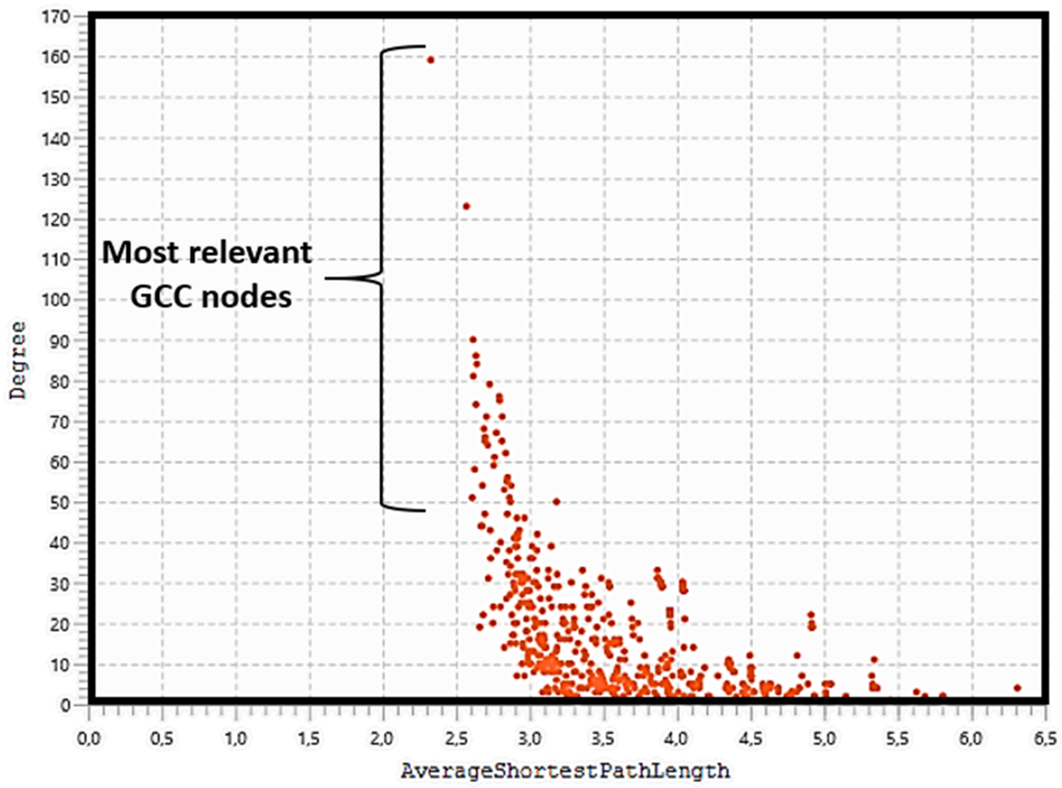

Figure S4 – Shortest path distribution - Distribution of the mean shortest paths as a function of the degree of the single nodes of the ORF7b Interactome. Calculation by Cytoscape through Centiscape [13]

The following list shows the first 30 proteins highlighted in figure 4S by the bracket as the most relevant GCC nodes. Many of these nodes, as we will see later, are important GCC functional nodes.

**Protein**	**Average Shortest Path Length**
EGFR	2.327272727272727
SRC	2.569090909090909
HSP90AA1	2.609090909090909
PIK3R1	2.6145454545454547
EGF	2.6163636363636362
AKT1	2.6254545454545455
PIK3CA	2.6345454545454547
SHC1	2.6345454545454547
GRB2	2.64

HSPA5	2.66
ESR1	2.6672727272727275
GNAI1	2.678181818181818
CTNNB1	2.678181818181818
HSPA8	2.6836363636363636
ERBB2	2.689090909090909
MAPK3	2.6945454545454544
STAT3	2.6945454545454544
MAPK1	2.6963636363636363
RHOA	2.7054545454545456
SOS1	2.7145454545454544
YWHAZ	2.7181818181818183
HRAS	2.727272727272727
PRKCA	2.730909090909091
CAV1	2.7345454545454544
SFN	2.749090909090909
MTOR	2.752727272727273
KRAS	2.7545454545454544
CDC42	2.76
PTPN11	2.772727272727273
GNAI2	2.7763636363636364

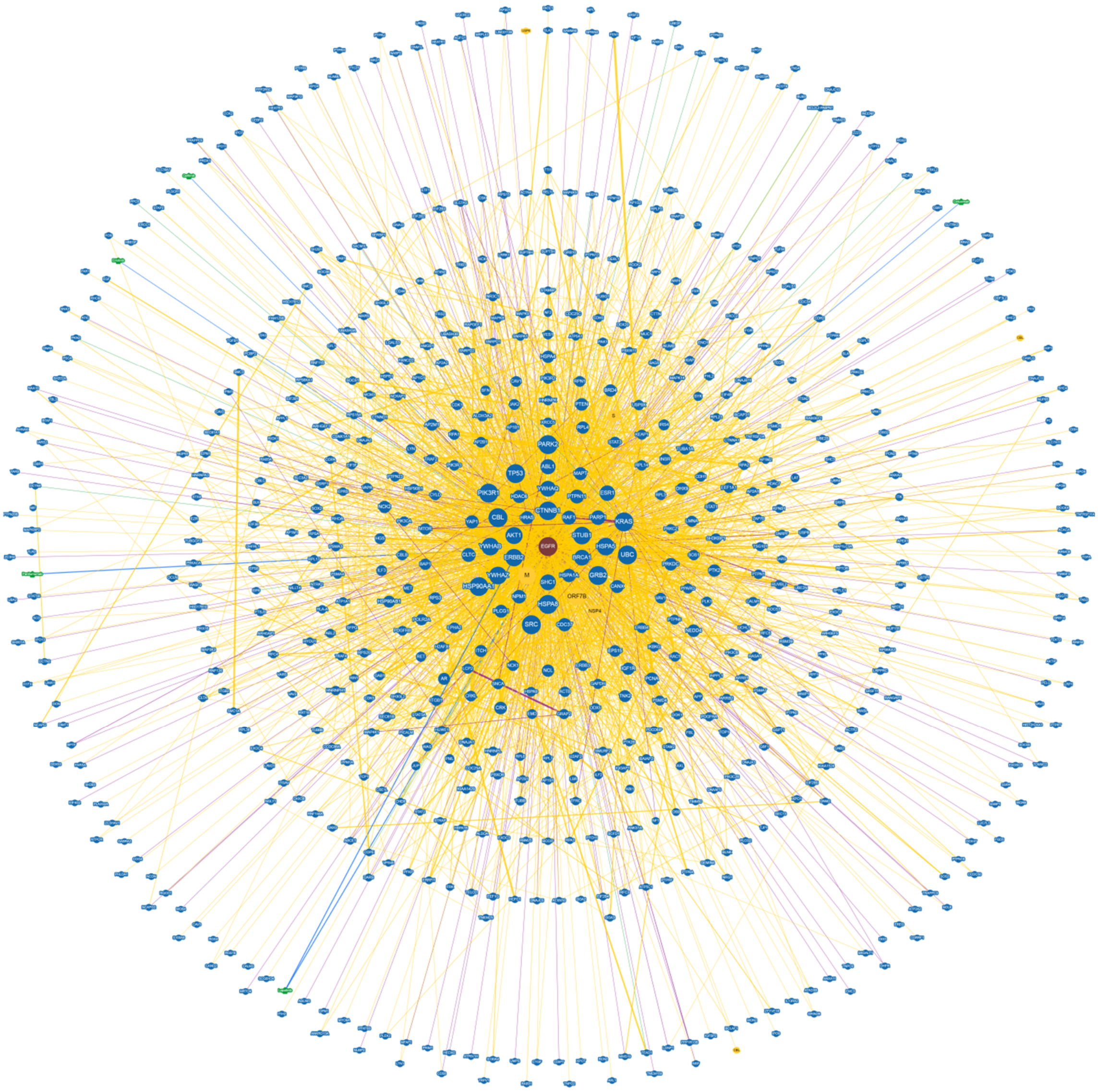

Figure S5 - EGFR interactome in the human proteome according to BioGRID. EGFR shows 3134 molecular interactors with 4897 interactions in the whole human proteome. Thus, EGFR has a huge potential to interact because of its 203 potential post-translational modification sites (PTMs). Few proteins in the human proteome possess such a feature. A single molecule can exhibit "modified forms" (or proteoforms) through a combinatorial pattern of co-occurring PTMs across multiple sites and a molecular population can exhibit a distribution of the amounts of the different forms. Proteoforms of the same protein are protein molecules chemically modified in one or more residues, therefore they cannot be considered structurally and functionally similar to the native protein encoded in the genome.

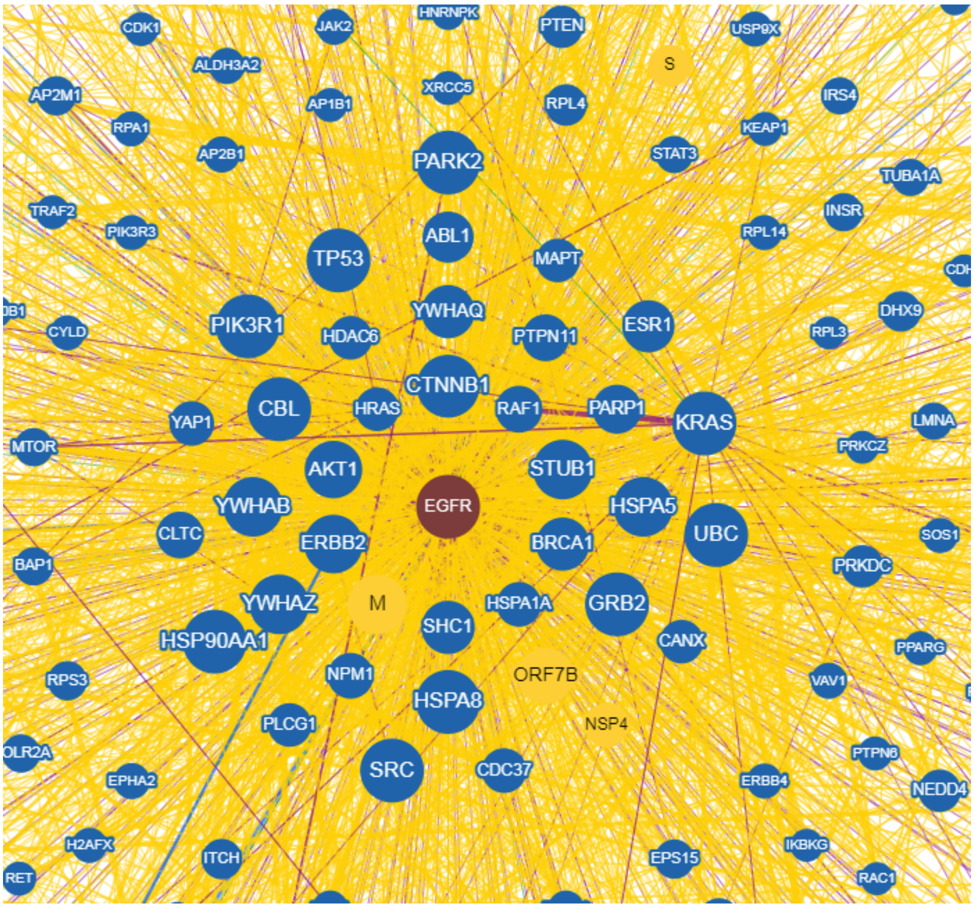

Figure S6 – Role of EGFR in the hub-and-spoke model - The figure is an enlargement of **fig 5S. It** shows the layers of higher degree nodes surrounding EGFR. We can also observe ORF7b, M, NSP4, and S of SARS-CoV-2 (in black and yellow). The figure shows the schematic of the hub-and-spoke model with its connections. Thus, the involved nodes are also present in the BioGRID interactome with a high level of statistical significance. While PIK3CA is in the BioGRID interactome, although with very little statistical significance, EGF is not present, but it came from the human proteome by enrichment. Hence, EGFR is an important metabolic multiplayer. It is a receptor of tyrosine kinase family that is activated by binding of its many ligands. This drives a series of intracellular signaling events that involve functional activations. All this makes us understand its enormous importance of EGFR in the viral strategy.

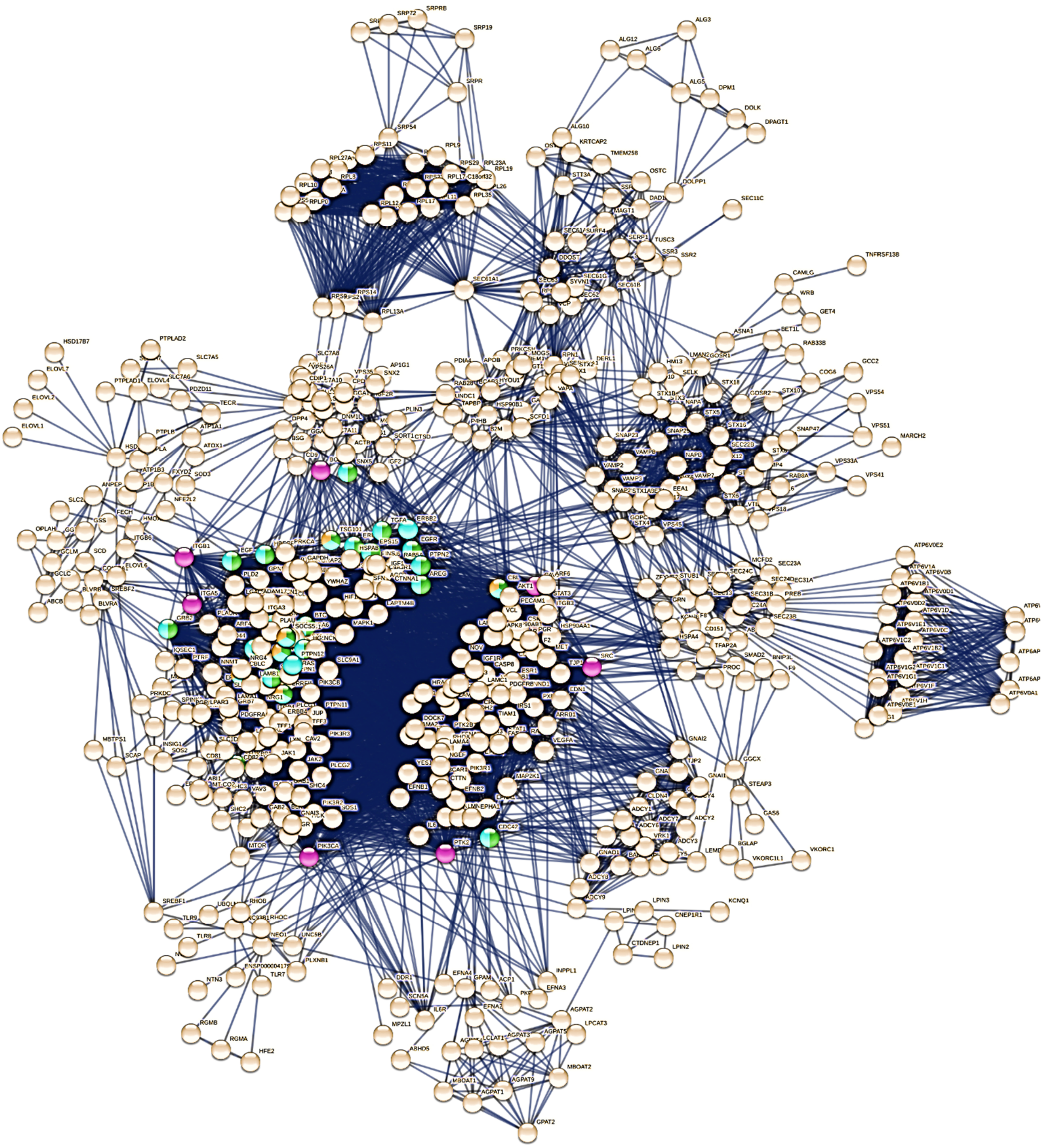

FIGURE S7 - Intensity of relationships among subgraphs. - The figure shows the intensity of the reciprocal relationships existing between the clusters. The strong relational density existing between clusters 1 and 9 is the highest in the network and shows the close functional interrelationships existing between these two metabolic modules. In the **figure 8S**, we show the enlarged central core to appreciate some interesting details.

By drawing upon polymer science, we can think about the viscoelastic physical hydrogels like cytosol made by a molecular network that relies on specific non-covalent bonding interactions. These networks are typically driven by enthalpy (negative ΔH° dominates), especially when the bonding pairs have a high level of structural complementarity and specificity [186,187]. When the temperature rises, the hydrogel's structural rigidity changes, causing its network to become looser and more dynamic.

In the cell, molecular networks are frequently based on long-distance, non-linear chaotic interactions, and the organism operates in isothermal conditions [188]. We know these interactions are physically present in biological networks [189-191] as dynamic and multivalent polymer-particle interactions.

Due to the high dynamism in transient networks, entropy (dominated by positive TΔS°) drives the networks, forming circuits driven by entropy. Such networks represent dynamic processes that not only affect the network's topology but also directly affect the process's evolution and its actions' dynamism. Moreover, according to Erwin Schrödinger, biological systems are first and foremost thermodynamic systems subject to negative variations in entropy capable of storing data and circulating signals that convey information. From a purely thermodynamic point of view, information is a negative change in entropy of a network to transport information [192].

Therefore, the physical foundation of biological networks, such as protein-protein networks, is independent of temperature because it is based on entropy rather than enthalpy [193,194]. The high relational density of the interactome network within the GCC core and between its two sub-graphs (clusters 1 and 9) suggests a functional activity with faster and more intense exchanges compared to the peripheral sub-graphs. Therefore, the importance of the GCC core of the interactome for understanding the ORF7b-induced effect cannot be underestimated. Moreover, many of the most important relational biomolecules are components of this core.

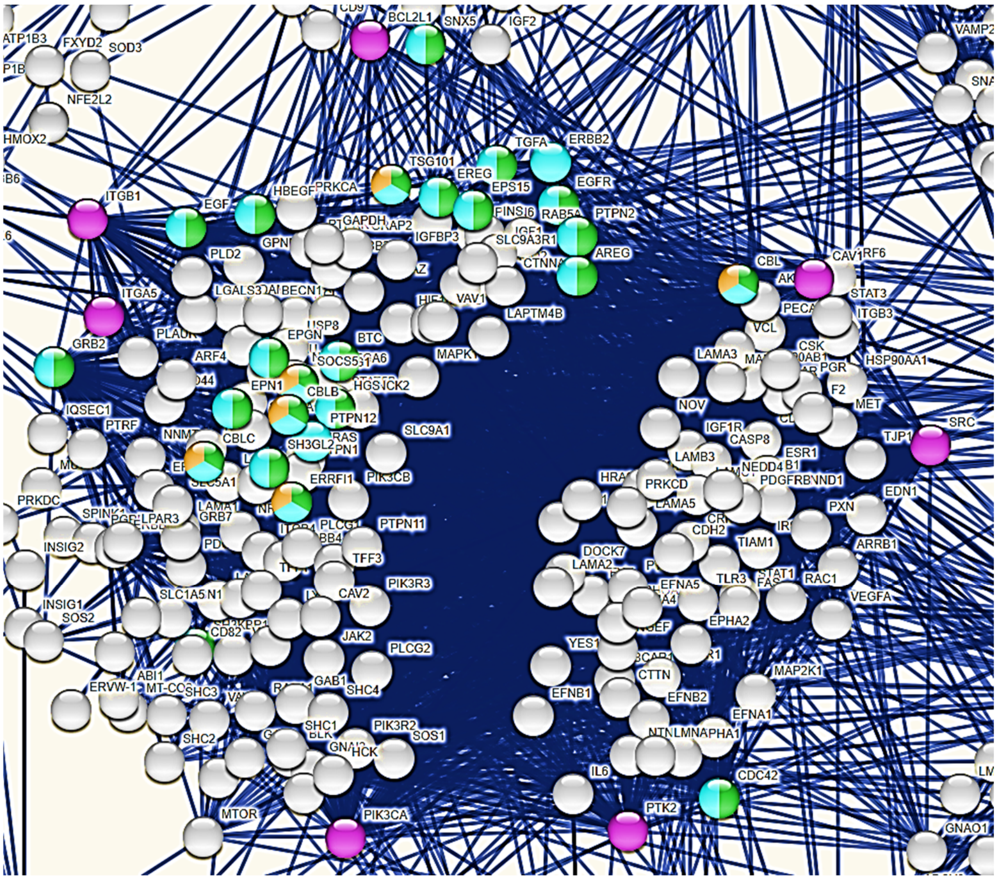

FIGURE S8 - Down-regulated processes of the core - This figure exemplifies four down-regulated processes of the core by showing the nodes involved according to the analysis by STRING in the table below. These

nodes concentrate in cluster 9. Of particular interest are the multiple roles assumed by many of them (presence of multiple colors) which shows their participation in multiple functional processes. Finally, we can see the nodes that down-regulate anoikis in violet color. They are not constitutive of a single module but take part in several modules and show strong functional relationships also with many of the peripheral modules. This shows how it is complex, if not impossible, to attribute a functional process to a set of nodes without evaluating them in the presence of the entire network.

GO:0042059	Negative regulation of epidermal growth factor receptor signali...	23 of 48	1.23	3.37e-17
GO:1901185	Negative regulation of ERBB signaling pathway	25 of 53	1.22	1.44e-18
GO:0007175	Negative regulation of epidermal growth factor-activated recept...	6 of 14	1.18	0.00017
GO:2000811	Negative regulation of anoikis	7 of 18	1.14	5.76e-05

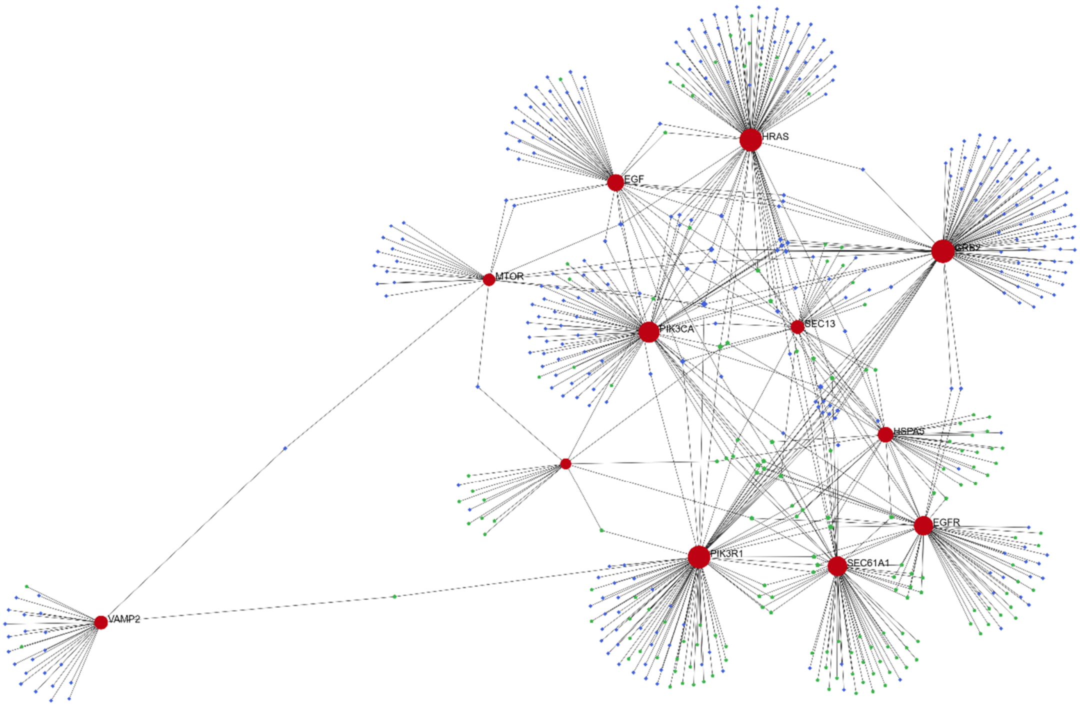

Figure S9 – Co-regulation network of high rank proteins - The figure shows a particular visual representation of a co-regulation network organized by NetworkAnalyst. The network shown refers to the network formed only by the previously isolated HUB and BOTTLENECK proteins (EGFR, HSPA5, MTOR, SEC13, SEC61A1, SRC, VAMP2, EGF, PIK3R1, PIK3CA, HRAS, GRB2). "Tufted interactions" are those determined by the sets of TFs and miRNAs specific to each node, while tiny, scattered nodes (always TFs and miRNAs) regulate the functional relationships between two or more core nodes. The network, as also shown in Figure 10S, follows the power law of biological networks.

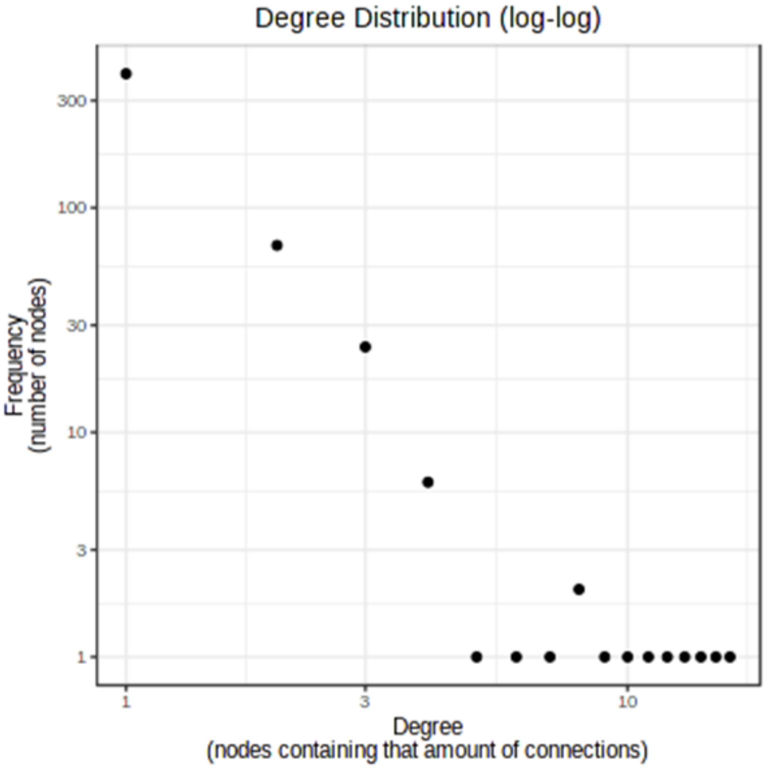

Figure S10 – Log-log distribution - This figure shows the characteristic log-log distribution of a scale-free network of nodes from the previous graph (**figure 9S**). Average path length: 3.82, radius: 3.0, diameter: 6.0, clustering coefficient: 0.16.

SECTION II

Robustness of the study

This work is based on interactions got from BioGRID. Although all 33,791 interactions have experimental origin, some of them are certainly random. The word random refers to those interactions that normally do not occur in the human organism simply because the two interacting proteins belong to totally different cellular compartments and, in vivo, the spatio-temporal possibility of an encounter hardly arises. Therefore, it was necessary to make a choice of the statistical levels of the molecular interactors selected for the study which, although subjected to a multi-step pruning process, could generate an interactome with distorted metabolic modules. The amount of the scientific basis defined by the literature articles selected by STRING, the characteristics of the protein associations, the intrinsic disorder among the proteins, which would support the randomness of the interactions, are all characteristics to be evaluated to show the data robustness.

We used as reference systems the values of the degree to select the suitable individual nodes.

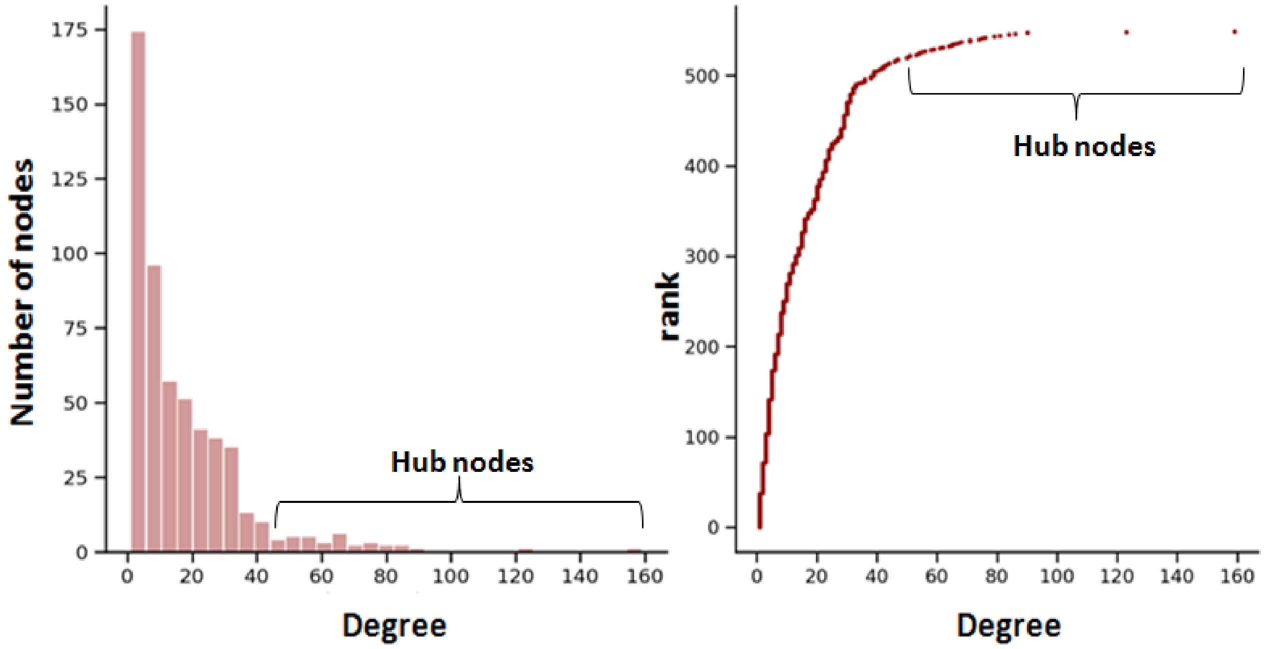

Figure S11 – Ranked values of nodes - Left graph - The histogram of interactome dataset. It shows the distribution of node values across the whole value range of degrees. The dataset comprises a substantial number of identical degree values that make up 92% of the whole dataset. The remaining nodes (44 nodes) are hub ones. **Right graph** - The graph charts the ranked values of nodes against their degree in the entire

dataset. The curve confirms that only about 8% of nodes get degree values above 40. However, also substantial part of the remaining nodes shows consistent high degree values. This also suggest very compact metabolic modules. There is no rule for defining the optimal number of hub nodes in a network. We relate this number to the number of protein modules and complexes that form the network. In this interactome, many compact complexes and modules require many nodes to determine functional stability. Calculation from STRING.

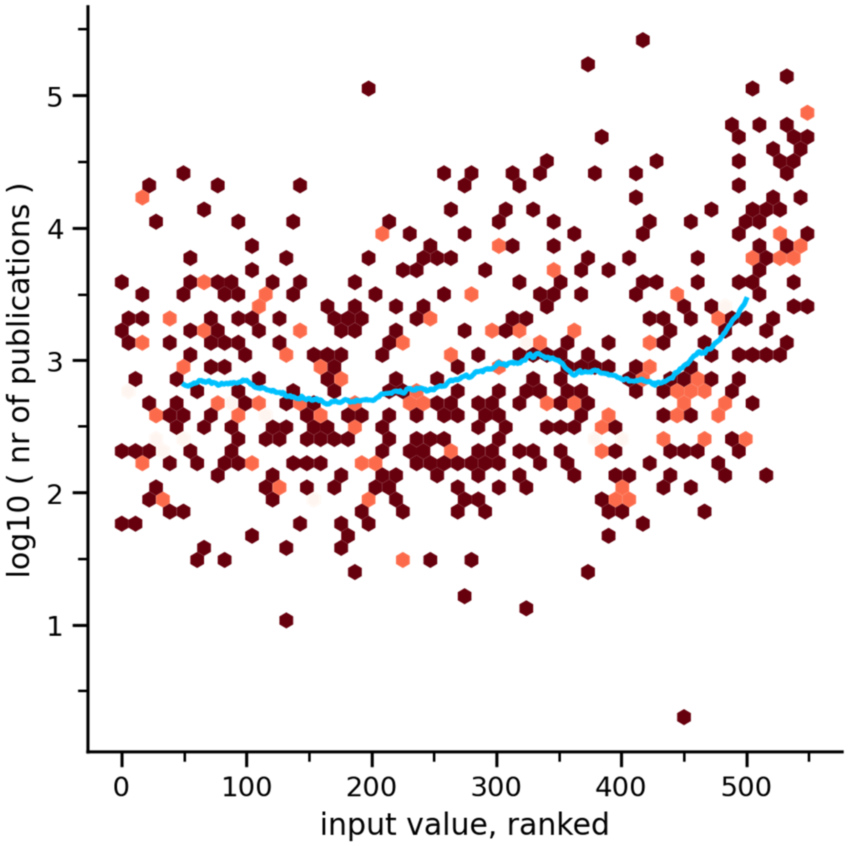

Figure S12 – Scientific literature distribution - The graph shows the relationship between ranked values for each protein and the number of the mentions of the associated proteins in the scientific literature. The tagged corpus of the STRING text-mining channel counts the number of publications that tag a given protein using at least one of its known names. This is what 'nr of publications' refer to. Proteins have been ranked from 1 to 551 according to their degree value. The proteins on the right have the highest degree values. Only a protein in the entire interactome (in the lower right corner), RPL17-C18orf32, a ribosomal subunit, does not possess a valuable number of scientific articles about association. Calculation from STRING.

Pearson's value: 0.274; Pearson's p-value: 6.44e-11; BP-R^2: 0.164 (very high). Statistical values have been calculated by STRING and the blue line delimits the average values. The significance of the graph is that the interactome statistically rests on robust experimental knowledge. The binned pseudo-R-Squared or BP-R2 is a measure (scale from 0 to 1) developed by Lun et al. [195] to quantify complex signaling relationships between two variables.

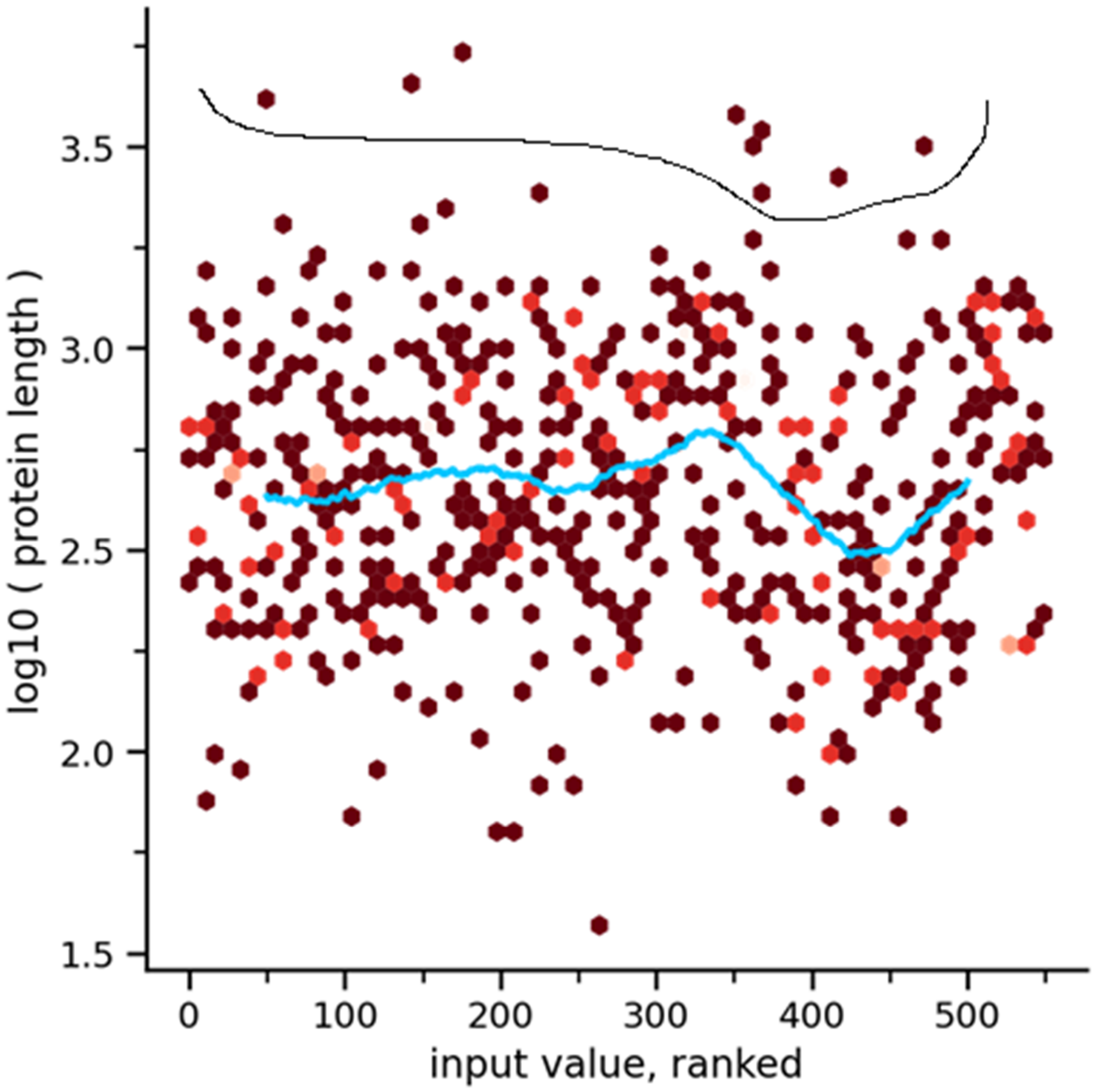

Figure S13 – Protein size distribution - This graph shows the relationship between degree values and the associated protein sizes with the blue line delimiting the average values. The graph shows many proteins of significant physical size, probably most involved in the formation of protein complexes. Calculation from STRING.

Pearson's r value: -0.003; Pearson's p-value: 0.9518; BP-R^2: 0.054 (medium).

The nine proteins that are in the top part of the graph with a log value > 3.30 (above the black line) are: MUC4 (Mucin, the major constituent of mucus, the viscous secretion that covers epithelial membrane surfaces); APOB (the main apolipoprotein of chylomicrons and large complex of Low-Density Lipoprotein (LDL), and it is also the ligand for the LDL receptor); PRKDC (DNA-dependent protein kinase (DNA-PK) [196]. It acts as a molecular sensor for DNA damage associated with the Polymerase II (PubMed:11955432, PubMed:12649176, PubMed:14734805, PubMed:33854234), and is also closely related to the establishment of central immune tolerance; LAMA1, 2, 3, 5, (members of the Laminin family, glycoproteins of the extracellular matrix, which are components of basal lamina); IGF2R, (Insulin Like Growth Factor 2 Receptor. This large receptor has various functions, including in the intracellular trafficking of lysosomal

enzymes, and the degradation of insulin-like growth factor 2); F8, (Coagulation Factor VIII, which takes part in the intrinsic pathway of blood coagulation); PLXNB1, (Plexin B1, plays a role in axon guidance, invasive growth and cell migration (PubMed:12198496), including negative regulation of cell adhesion and of cell shape. Is integral component of plasma membrane); DOCK7, Dedicator of cytokinesis protein 7, shows many functions including neuronal polarization (PubMed:16982419), regulate the actin cytoskeleton (PubMed:29467281), and is involved in regulating cortical neurogenesis.

All these proteins are prone to the formation of proteic complexes or aggregations, often acting as a scaffold.

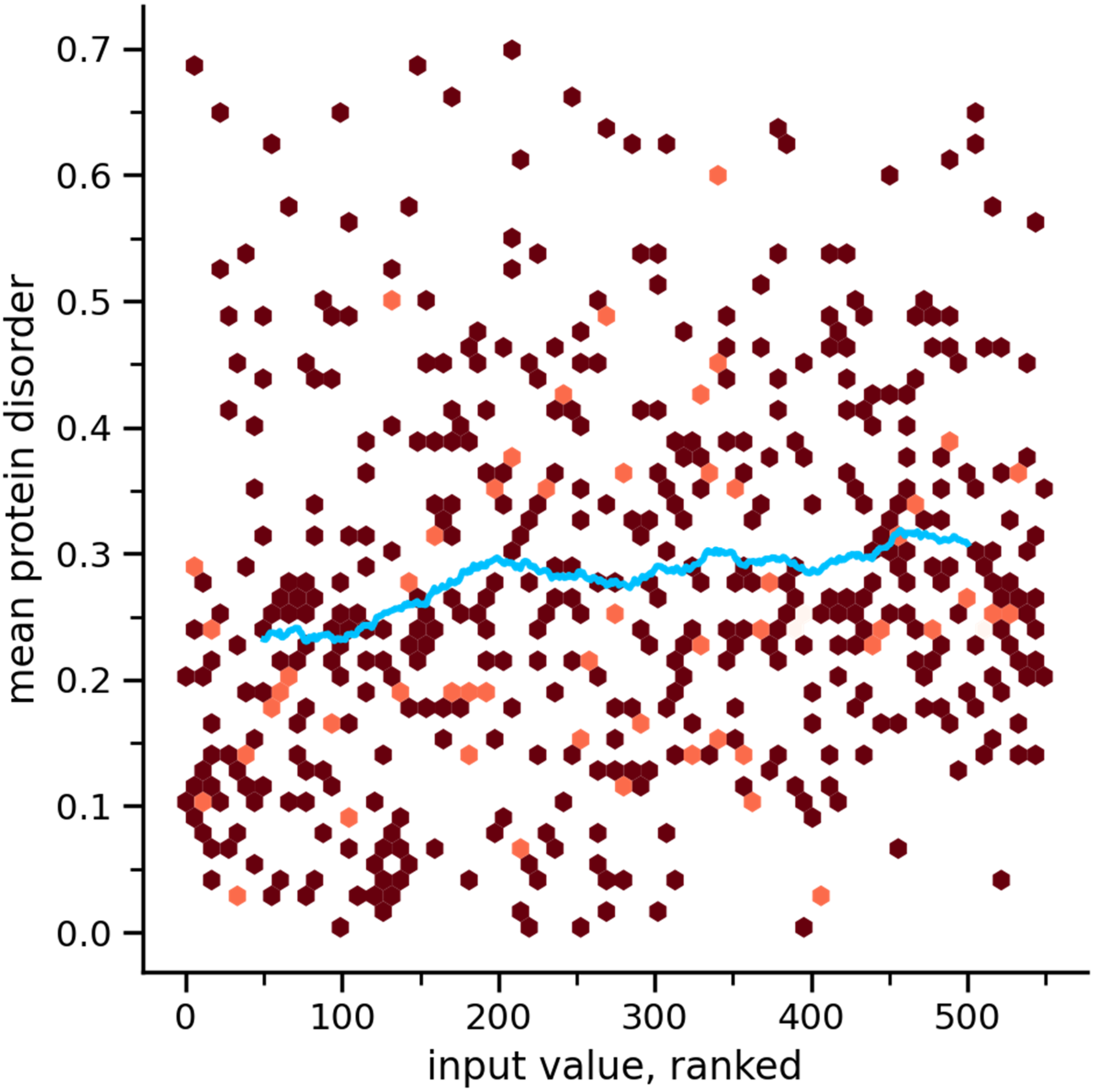

Figure S14 – Protein disorder distribution - The graph shows the relationship between the ranked values and the full-length average protein disorder of the associated proteins. Even if the 3D structure provides a complete information for PPI prediction, with the emergence of the intrinsically disordered proteins [91] and the induced fit theory [197], the disorder becomes a crucial information for PPI computational evaluation. Calculation from STRING.

In the graph, the intrinsic disorder level is on average low (most proteins have less than 30% of intrinsic disorder). These data show that most of the interactions do not exploit disordered structural segments of the interacting proteins, but interactions take place because of the presence of specific structural interaction sites, evolutionarily programmed. Therefore, we are dealing with a rather small number of random interactions. It is in fact known that intrinsically disordered proteins have a great tendency to interact, structurally adapting their conformation to the target protein. The situation is different for viral proteins that have not had the evolutionary time to adapt their structure to human proteins (further details in "Results").

The blue line delimits the average values. Pearson's r value: 0.167; Pearson's p-value: 8.46e-05; BP-R^2: 0.034 (medium). These observations, together with the results got from the interactome, support the validity of the experimental design used, which is to be placed on a perimeter of knowledge with consistent statistical validity. All calculations were performed using the services made available by STRING.

These observations, together with the results obtained from the interactome, support the validity of the experimental design used, which is to be placed on the perimeter of the present knowledge and characterized by consistent statistical validity. We performed these calculations using the services made available by STRING.
